# Metagenomic next-generation sequencing for detecting lower respiratory tract infections in sputum and bronchoalveolar lavage fluid samples from children

**DOI:** 10.3389/fcimb.2023.1228631

**Published:** 2023-08-17

**Authors:** Ruihe Shi, Yuan Wang, Shujuan Zhou, Yanli Zhang, Shiwei Zheng, Dingfang Zhang, Xilong Du, Weiyue Gu, Yiran Xu, Changlian Zhu

**Affiliations:** ^1^ Division of Pulmonology, Department of Pediatrics, Third Affiliated Hospital of Zhengzhou University, Zhengzhou, China; ^2^ Henan Pediatric Clinical Research Center and Key Laboratory of Child Brain Injury, Third Affiliated Hospital and Institute of Neuroscience of Zhengzhou University, Zhengzhou, China; ^3^ Department of Clinical Laboratory, Third Affiliated Hospital of Zhengzhou University, Zhengzhou, China; ^4^ Department of Biochemistry and Molecular Biology, College of Life Sciences, Xiamen University, Xiamen, China; ^5^ Department of Pediatrics, People’s Hospital of Xiping County, Zhumadian, Henan, China; ^6^ Department of Pediatrics, People’s Hospital of Biyang County, Zhumadian, Henan, China; ^7^ Beijing Chigene Translational Medical Research Center, Beijing, China; ^8^ Center for Brain Repair and Rehabilitation, Institute of Neuroscience and Physiology, University of Gothenburg, Gothenburg, Sweden

**Keywords:** metagenomic next-generation sequencing, bronchoalveolar lavage fluid, sputum, pediatric, lower respiratory tract infection

## Abstract

Lower respiratory tract infections are common in children. Bronchoalveolar lavage fluid has long been established as the best biological sample for detecting respiratory tract infections; however, it is not easily collected in children. Sputum may be used as an alternative yet its diagnostic accuracy remains controversial. Therefore, this study sought to evaluate the diagnostic accuracy of sputum for detecting lower respiratory tract infections using metagenomic next-generation sequencing. Paired sputum and bronchoalveolar lavage fluid samples were obtained from 68 patients; pathogens were detected in 67 sputum samples and 64 bronchoalveolar lavage fluid samples by metagenomic next-generation sequencing, respectively. The combined pathogen-detection rates in the sputum and bronchoalveolar lavage fluid samples were 80.90% and 66.2%, respectively. For sputum, the positive predictive values (PPVs) and negative predictive values (NPVs) for detecting bacteria were 0.72 and 0.73, respectively, with poor Kappa agreement (0.30; 95% confidence interval: 0.218–0.578, *P* < 0.001). However, viral detection in sputum had good sensitivity (0.87), fair specificity (0.57), and moderate Kappa agreement (0.46; 95% confidence interval: 0.231–0.693, *P* < 0.001). The PPVs and NPVs for viral detection in sputum were 0.82 and 0.67, respectively. The consistency between the sputum and bronchoalveolar lavage fluid was poor for bacterial detection yet moderate for viral detection. Thus, clinicians should be cautious when interpreting the results of sputum in suspected cases of lower respiratory tract infections, particularly with regards to bacterial detection in sputum. Viral detection in sputum appears to be more reliable; however, clinicians must still use comprehensive clinical judgment.

## Introduction

Lower respiratory tract infections (LRTIs) are the most common infectious causes of death globally, whereof pneumonia is the primary cause of death in children under five years of age (Bryce et al., 2005). Bacteria, viruses, mycoplasma, fungi, and parasites cause LRTIs which each require distinct infection control strategies and therapies. Pathogen-specific therapy is the most effective technique to cure pneumonia. However, in the absence of an identifiable cause, doctors often administer empirical medications based on experience which may easily lead to antimicrobial overuse and the spread of antibiotic-resistant bacteria.

Pathogen identification in bronchoalveolar lavage fluid (BALF) is the gold standard for lower respiratory tract infections; unfortunately, BALF must be retrieved during bronchoscopy which is typically conducted under anesthesia. Therefore, it is reserved for patients with complicated or severe illnesses. Indications include those patients who are admitted to intensive care units, immunocompromised children, and children with severe and recurrent respiratory symptoms (Wurzel et al., 2013). In contrast, sputum is often the specimen of choice for identifying pathogens in children with LRTIs due to its accessibility, simplicity, and lower risks. Sputum analysis results affect the choice of therapy, particularly antimicrobial (Zar et al., 2012). However, whether pathogen detection in sputum is an accurate representation of the causative pathogen of the LRTI remains controversial.

In clinical practice and research, rapid and accurate pathogen identification is essential. Despite the availability of several detection techniques, pathogen identification remains challenging. Culture - the gold standard for microbiological identification - is time-consuming, has limited sensitivity, particularly for fastidious species, and is readily influenced by antimicrobial use. Furthermore, the culture process only applies to bacteria and fungi; viruses cannot be cultivated in the absence of a suitable host (Li et al., 2018; Chen et al., 2020). Although culture-independent approaches, such as immunological tests and nucleic acid testing utilizing PCR, are fast and precise, previous information or assumptions about the species of the pathogenic microorganisms are required. Metagenomic next-generation sequencing (mNGS) uses unbiased sampling and permits the simultaneous detection of all potentially infectious organisms; thus, it may serve as a breakthrough diagnostic tool capable of overcoming the limitations of existing methods (Wang et al., 2019; Diao et al., 2022). Therefore, this study sought to evaluate and compare the diagnostic performance of mNGS for detecting pathogens (bacteria, fungi, and viruses) in children with LRTIs in BALF and sputum specimens.

## Materials and methods

### Study design and patient selection

From September 1, 2020 to March 31, 2021, we prospectively included pediatric patients with LRTIs treated at The Third Affiliated Hospital of Zhengzhou University. Specific diagnostic criteria for LRTIs included the presence of fever; respiratory symptoms, such as cough, shortness of breath, wheezing, or rattling chest; and suggestive features on chest X-ray or CT scan. The study comprised 68 hospitalized patients with LRTI treated for at least 72 hours without clinical improvement, or persistent elevated infectious markers in blood or deterioration on chest radiographs. All participants fulfilled the indications to undergo bronchoscopy (Experts Group of Pediatric Respiratory Endoscopy et al., 2018). After obtaining written informed consent from the participants’ legal guardians to undergo the procedure, BALF collection was performed using bronchoscopy. The prospective cohort study design was approved by the ethics committee of The Third Affiliated Hospital of Zhengzhou University (Ethics approval no. 2021-058-01). Written informed consent was obtained from the legal guardians of all participants prior to enrolment in the study.

**Figure 1:**
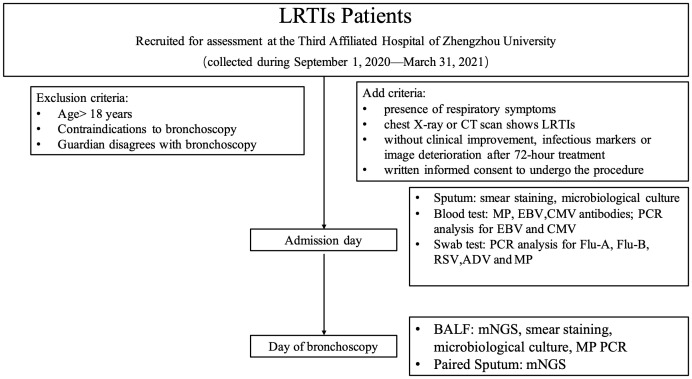
Study design. MP, *M. pneumoniae*; EBV, Epstein-Barr virus; CMV: cytomegalovirus; Flu, influenza; RSV, respiratory syncytial virus; ADV, adenovirus; mNGS, metagenomic next-generation sequencing.

All participants had undergone conventional microbiological testing upon admission, including blood sampling for antibody detection against *Mycoplasma pneumoniae*, Epstein-Barr virus (EBV), and cytomegalovirus (CMV); PCR analysis for EBV and CMV; and pharyngeal swabs for influenza A, influenza B, adenovirus (ADV), respiratory syncytial virus (RSV), and *M. pneumoniae* for PCR analyses. Sputum specimens were preserved for smear microscopy, staining, and microbiological culture. Expectorated sputum samples were obtained from children who could expectorate. An attendant nurse collected sputum samples using nasotracheal suctioning from children who could not expectorate. Senior clinical laboratories conducted and interpreted the Gram stain. Samples were assessed to be of good quality if each low power field included at least 25 polymorphonuclear cells and 10 squamous epithelial cells. Samples were otherwise considered to be of low quality. Bronchoscopies were performed by trained bronchoscopists according to standard protocols in patients with suitable indications. Paired sputum and BALF specimens were collected contemporaneously to undergo mNGS testing. Additionally, the BALF was routinely tested for microbiological smears, cultures, and *M. pneumoniae* by PCR (**Figure 1**). The paired sputum and BALF specimens for mNGS testing were stored at −80°C from within one hour after collection. Each sample was analyzed for DNA and RNA using DNBSEQ-T7 platforms (MGI Tech Co., Ltd, Shenzhen, China) with 150 bp paired-end mode.

### Conventional microbiological testing methods

*Mycoplasma pneumoniae*, EBV and CMV antibodies were detected by enzyme-linked immunosorbent assay using a detection kit (Beijing Beier Bioengineering Co., Ltd., Beijing, China). PCR analysis for CMV, EBV and *M. pneumoniae* were performed using a kit from Amplly Biotech (Xiamen Amplly Biotechnology Co., Xiamen, China). Using GENESIS antigen kits (Hangzhou Genesis Biodetection & Biocontrol Co., Ltd, Hangzhou, Zhejiang, China), influenza A, influenza B, ADV, and RSV were detected. Gram-stain was determined by using the BaSO Gram-staining kit (BaSO Medical Devices Co., Ltd., New Taipei City, Taiwan). Specimens were sent to the laboratory for screening of pathogens using semiquantitative culture (Autobio Diagnostics Co., Zhengzhou, China) and to determine their antimicrobial susceptibility using VITEK^®^2 (BioMérieux, Craponne, France).

### mNGS and analysis

According to the manufacturer’s instructions, DNA/RNA was extracted from 200–300 μL sputum and BALF samples using the TIANMicrobe Magnetic Patho-DNA/RNA Kit (TIANGEN Biotech Co., Ltd., Beijing, China). The extracted DNA/RNA samples were subjected to quality inspection by Qubit 4.0 Fluorometer/NanoDrop One (Thermo Fisher Scientific Inc., Waltham, MA, USA). The cutoff concentration of DNA ≥ 0.05 ng/μL and the A260/A280 of RNA was 1.9–2.1. The next analysis was continued after quality confirmation. RNA was reverse-transcribed into full-length cDNA using the Full-length cDNA Synthesis Kit (YEASON, Shanghai, China). The reverse-transcribed cDNA and DNA were used for library construction using the Universal Plus DNA Library Prep Kit for MGI V2 (Vazyme Biotech Co., Ltd., Nanjing, China). The constructed library was subjected to quality inspection by the Qubit 4.0 Fluorometer (Thermo Fisher Scientific Inc., US) and Qsep400 fragment analyzer (BiOptic BIO-TECH Inc., Jiangsu, China). High-throughput sequencing through the DNBSEQ-T7 sequencing platform (MGI Tech Co., Ltd., Shenzhen, China) was then completed by the Chigene Translational Medical Research Center Co. Ltd. (Beijing, China).

1. Data Quality Control: We first performed quality control on the raw sequencing data by removing low-quality bases and adapters using Trimmomatic (version 0.39). The data volume, Q30, and GC content were then calculated. If the number of clean data reads was less than 10 million, the sample was flagged for retesting. Additionally, if Q30 was less than 80% or GC content was less than 30%, the sample was subjected to further experimentation.
2. Host Removal: After quality control, we removed human sequences using snap-aligner (version 1.0.20) and bowtie2 (version 2.4.4) alignment software. Ribosomal rRNA sequences were removed using SortMeRNA (version 4.3.4). We then calculated the proportion of non-host sequences.
3. Species Identification, Filtering, Annotation, and Genome Coverage Calculation:
a. After obtaining relatively clean non-host sequences, we performed BLASTn (version 2.11.0) alignment to identify the species information for the non-host sequences.
b. For sequences that matched the pathogenic microorganism database, we classified them into two groups based on coverage and identity. Sequences with coverage ≥95% and identity ≥90% were classified as Group A, and those with coverage ≥85% and identity ≥85% were classified as Group B. We performed LCA filtering for Group A to select species-specific sequences, counted the number of sequences for each species, and annotated the species in the knowledge base. For Group B, we calculated the genome coverage for each species.
c. We then applied a filtering rule for species belonging to the same genus, as follows:
i. If a genus had only one species, that species passed the filtering rule, and its ‘genus_pass’ flag was set to 1.
ii. If the most abundant species accounted for more than 75% of the total reads in a genus, only that species passed the filtering rule, with ‘genus_pass’ set to 1, while other species did not pass, and their ‘genus_pass’ was set to 0.
iii. If the most abundant species accounted for more than 40% of the total reads in a genus, and the second most abundant species accounted for more than 30%, both species passed the filtering rule, with ‘genus_pass’ set to 1, while other species did not pass, and their ‘genus_pass’ was set to 0.
iv. If none of the above conditions were met, all species in that genus passed the same genus filtering rule, with ‘genus_pass’ set to 1.
4. Summarizing Species Annotation Results and Generating Report Data: We combined the species annotation results and generated a comprehensive report, which included information on the identified species, their relative abundance, genome coverage, and annotations from the knowledge base.

### Data collection

During hospitalization, baseline characteristics such as name, age, sex, symptoms, underlying diseases, medication history, and epidemiological history were collected. Furthermore, data such as laboratory test results (e.g., blood routine and biochemical tests), imaging findings, and treatments (e.g., antimicrobial) used, were obtained. Clinical outcomes were also recorded, including hospital stay duration and overall medical expenditure.

### Statistical analyses

Enumeration data were displayed as numbers and percentages, whereas measurement data were shown as means with standard deviations or medians (ranges). We used 2 × 2 contingency tables to ascertain the PPVs, NPVs, sensitivity, and specificity of mNGS in sputum and BALF samples for patients with paired findings. A Kappa analysis was used to measure the degree of agreement between sampling methodologies. A two-tailed *P*-value of less than 0.05 was deemed statistically significant. SPSS V20.0 was used for the statistical analyses (IBM SPSS Statistics for Windows, IBM Corp., Armonk, NY, USA).

## Results

### Baseline characteristics

This study included 68 pediatric patients with LRTIs consisting of 47 males (69.1%) and 21 females (30.9%). The median age was one year three months (range: 1 month to 11 years); most were younger than three years (70.6%). Four patients had tracheomalacia, six had airway stenosis, and twelve had congenital heart disease. Foreign bodies were found in the trachea of eight individuals. Eleven individuals had a vitamin D deficiency, and eight patients were anemic. Furthermore, one patient had a skin fistula, one had hydronephrosis, and one had a subglottic hemangioma. The average time from admission to sampling was three days (range: 3 to 20 days), and all patients were treated with antibiotics before bronchoscopy (**Table 1**).

**Table 1:** Baseline data characteristics of children with LRTIs.

Characteristic	Patient data, n (%)
Sex (%)	
Male	47 (69.1%)
Female	21 (30.9%)
Age (years old)	
Median (range) age	1y3m (1mo to 11 y r)
≤1	27
1—3	21
3—5	13
>5	7
Underlying disease (%)	
Congenital heart disease	12 (17.6%)
Tracheomalacia	4 (5.9%)
Tracheal stenosis	6 (8.8%)
Tracheal foreign body combined with pulmonary infection	8 (11.7%)
Anemia	6 (8.8%)
Vitamin D deficiency	11 (16.2%)
Cutaneous fistula	1 (1.5%)
Hydronephrosis	1 (1.5%)
Subglottic hemangioma	1 (1.5%)
Days from admission to sampling	3(1,4)

### Pathogen detection by mNGS in paired BALF and sputum samples

Overall, 64 (94.1%) and 67 (98.5%) patients were pathogen-positive by mNGS in the paired BALF and sputum specimens, respectively (*P* = 0.366). In the BALF samples, 19 (27.9%) cases were found to have a single pathogen, and 45 (66.2%) had a mix of pathogens. However, for the paired sputum samples, 12 (17.6%) cases had a single pathogen, and 55 (80.9%) cases had a mix of pathogens.

**Figure 2:**
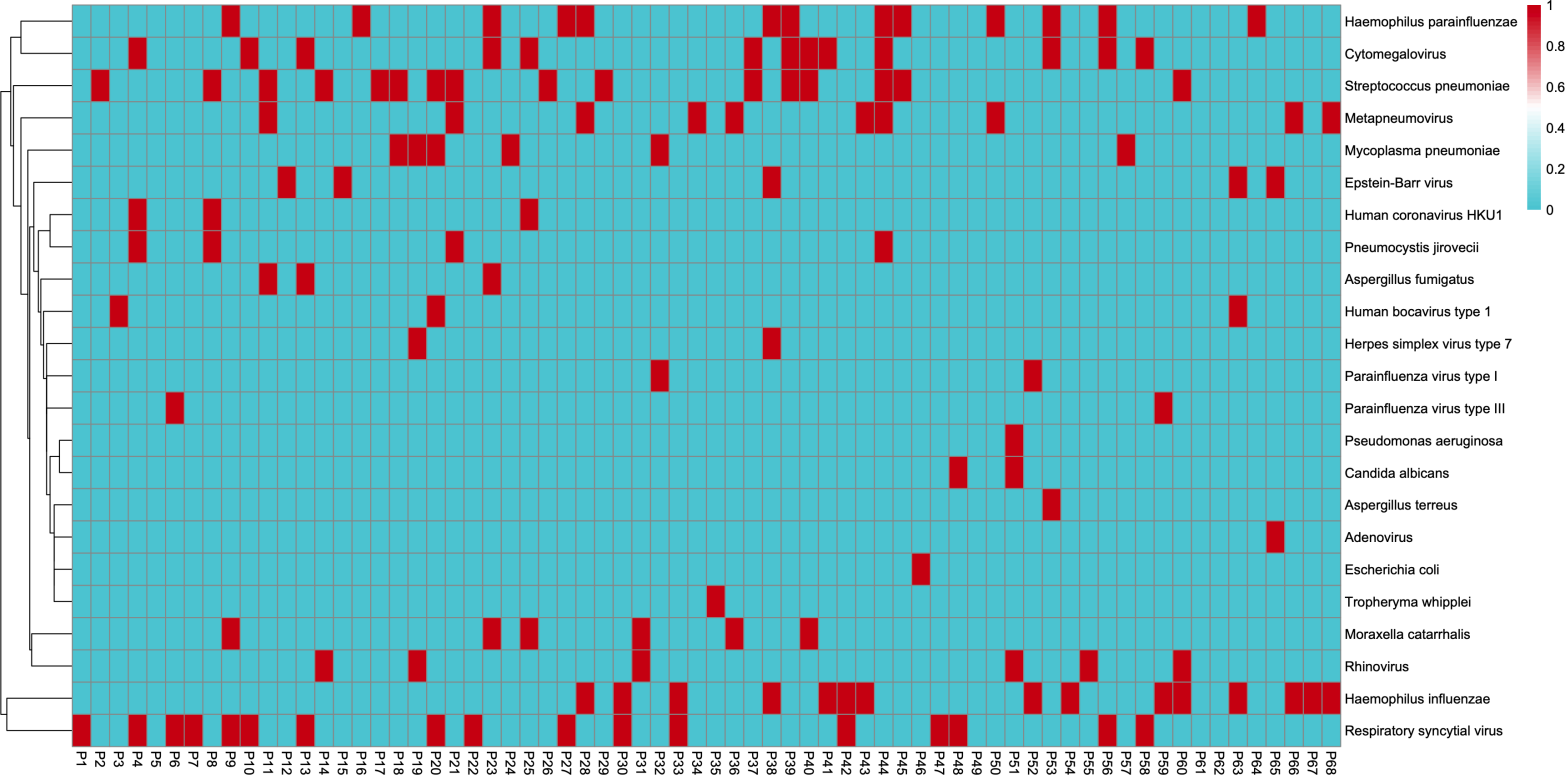
Metagenomic next-generation sequencing results from bronchoalveolar lavage fluid samples. The horizontal coordinates represent each patient from 1 to 68. The vertical coordinates represent positive (red) and negative (blue) results for pathogen detection.

In the BALF samples and the paired sputum specimens, the most common bacterial species and mycoplasma were (in descending order): *Streptococcus pneumoniae*, *Haemophilus influenzae*, *Haemophilus parainfluenzae*, *Moraxella catarrhalis*, and *M. pneumoniae*. In descending order, the most common viruses in the BALF samples were RSV, CMV, metapneumovirus, rhinovirus, and EBV, while in the sputum samples, the most common viruses were RSV, CMV, rhinovirus, metapneumovirus, and EBV. Additionally, three fungi were detected: *Pneumocystis jirovecii* (BALF: 4 cases; sputum: 3 cases), *Aspergillus fumigatus* (BALF: 3 cases; sputum: 0 cases), and *Candida albicans* (BALF: 2 cases; sputum: 2 cases) (**Figure 2**).

### Consistency of mNGS pathogen detection in paired BALF and sputum samples

Using the BALF pathogen results as the reference, we calculated the sensitivity, specificity, PPV, NPV, and Kappa agreement value of the sputum pathogen results, which are presented in **Table 2**.

**Table 2:** A comparative detection of pathogens by mNGS from BALF and paired sputum samples in LRTI patients.

Pathogenic species	BALF mNGS+	Sputum mNGS+	Sputum+/ BALF+	Sputum – /BALF+	Sputum+/ BALF–	Sputum–/ BALF–	PPV	NPV	Se	Sp	Kappa
**Bacteria**	44	57	41	3	16	8	0.72	0.73	0.93	0.33	0.30
*Streptococcus pneumoniae*	16	26	14	2	12	40	0.54	0.95	0.88	0.77	0.53
*Haemophilus influenzae*	15	20	14	1	6	47	0.70	0.98	0.93	0.89	0.73
*Haemophilus parainfluenzae*	13	11	7	6	4	51	0.64	0.89	0.54	0.93	0.50
*Moraxella catarrhalis*	6	7	2	4	5	57	0.29	0.93	0.33	0.92	0.24
*Escherichia coli*	1	1	1	0	0	67	–	–	–	–	–
*Pseudomonas aeruginosa*	1	0	0	1	0	67	–	–	–	–	–
*Tropheryma whipplei*	1	0	0	1	0	67	–	–	–	–	–
*Staphylococcus aureus*	0	3	0	0	3	65	–	–	–	–	–
**Virus**	47	50	41	6	9	12	0.82	0.67	0.87	0.57	0.46
RSV	17	16	14	3	2	49	0.88	0.94	0.82	0.96	0.80
CMV	13	12	10	3	2	53	0.83	0.95	0.77	0.96	0.76
hMPV	10	10	8	2	2	56	0.80	0.97	0.80	0.97	0.77
HRV	6	11	6	0	5	57	0.55	1.00	1.00	0.92	0.67
EBV	5	3	1	4	2	61	–	–	–	–	–
HBoV-1	3	7	2	1	5	60	–	–	–	–	–
HCoV-HKU1	3	4	2	1	2	63	–	–	–	–	–
HPIV-III	2	5	1	1	4	62	–	–	–	–	–
HPIV-I	2	1	1	1	0	66	–	–	–	–	–
Adenovirus	1	3	1	0	2	65	–	–	–	–	–
HCoV-NL63	0	3	0	0	3	65	–	–	–	–	–
HSV-1	0	2	0	0	2	66	–	–	–	–	–
HSV-7	2	0	0	2	0	66	–	–	–	–	–
**MP**	6	6	6	0	0	62	1.00	1.00	1.00	1.00	1.00
**Fungi**	10	5	5	5	0	58	–	–	–	–	–
PJ	4	3	3	1	0	64	1.00	0.98	0.75	1.00	0.85
*Aspergillus fumigatus*	3	0	0	3	0	65	–	–	–	–	–
*Candida albicans*	2	2	2	0	0	66	–	–	–	–	–
Aspergillus terreus	1	0	0	1	0	67	–	–	–	–	–

BALF as the arbitrary gold-standard.

BALF, bronchoalveolar lavage fluid; RSV, respiratory syncytial virus; CMV: Cytomegalovirus; hMPV, human metapneumovirus; EBV, Epstein-Barr virus; HRV, human rhinovirus; HBoV, Human Bocavirus; HCoV, human coronavirus; HPIV, human para-influenza virus; HSV, herpes simplex virus; MP, mycoplasma pneumoniae; PJ, pneumocystis jirovecii; PPV, positive predictive value; NPV, negative predictive value; Se, sensitivity; Sp, specificity.

+ means positive; – means negative.

For bacterial detection in sputum, the overall sensitivity, specificity, PPV, and NPV were 0.93, 0.33, 0.72 and 0.73, respectively. The Kappa value was 0.30 (95% confidence interval [CI]: 0.218–0.578, *P* < 0.001). However, various microorganisms yielded different outcomes. *H. influenzae* had the highest Kappa value (0.73) of the four most common bacteria, while *M. catarrhalis* had the lowest (0.24). Statistical analyses were not performed for the remaining four bacterial species with low detection rates due to the limited number of cases. Additionally, one case of *Escherichia coli* was identified in the sputum and BALF of the same patient. Furthermore, one case of *Pseudomonas aeruginosa* and one case of *Tropheryma whipplei* were detected in the BALF samples, although neither was detected in the sputum samples. Finally, three instances of *Staphylococcus aureus* were identified in the sputum, but none were detected in the BALF.

For viral detection in sputum, the sensitivity, specificity, PPV, and NPV were 0.87, 0.57, 0.82, and 0.67, respectively. The Kappa value was 0.46 (95% CI: 0.231–0.693, *P* < 0.001), slightly higher than that for bacteria. The Kappa value for RSV was 0.80, indicating a very high level of consistency. The Kappa values for CMV, metapneumovirus, and rhinovirus, which had high detection rates, were 0.76, 0.77, and 0.67, respectively. Six patients tested positive for *M. pneumoniae* on both the BALF and sputum samples. Furthermore, *C. albicans* was detected in two patients’ BALF and sputum samples, and *A. fumigatus* was detected in the BALF of three individuals but not identified in the sputum. Finally, *P. jirovecii* was detected in the BALF of four cases, of which three were also positive in the sputum.

### Conventional culturing versus mNGS for bacterial detection in BALF

The conventional microbial cultures of BALF samples from 68 patients yielded positive bacterial results in 12 individuals (positivity rate: 17.6%) with five types of single-pathogen bacteria. However, 44 patients tested positive for bacteria using mNGS (positivity rate: 64.7%), demonstrating a statistically significant difference from the conventional method (*P* < 0.01). Of the 12 patients with positive conventional bacterial cultures, one patient had *Enterobacter cloacae* detected on culture yet no bacteria were detected by mNGS, one patient had a culture result of *S. pneumoniae* yet mNGS detected *H. influenzae*, and the remaining 10 patients had the same pathogenic bacteria detected by mNGS. (**Table 3**).

**Table 3:** The distinction between the conventional culture approach and mNGS for BALF bacterial detection.

Patient Number	BALF-mNGS	BALF-conventional culture
2	*Streptococcus pneumoniae*	–
8	*Streptococcus pneumoniae*	–
9	*Moraxella catarrhalis* *Haemophilus parainfluenzae*	*Moraxella catarrhalis*
11	*Streptococcus pneumoniae*	*Streptococcus pneumoniae*
14	*Streptococcus pneumoniae*	–
16	*Haemophilus parainfluenzae*	–
17	*Streptococcus pneumoniae*	–
18	*Streptococcus pneumoniae*	–
20	*Streptococcus pneumoniae*	–
21	*Streptococcus pneumoniae*	–
23	*Moraxella catarrhalis* *Haemophilus parainfluenzae*	–
25	*Moraxella catarrhalis*	–
26	*Streptococcus pneumoniae*	–
27	*Haemophilus parainfluenzae*	–
28	*Haemophilus parainfluenzae* *Haemophilus influenzae*	–
29	*Streptococcus pneumoniae*	–
30	*Haemophilus influenzae*	–
31	*Moraxella catarrhalis*	*Moraxella catarrhalis*
33	*Haemophilus influenzae*	–
35	*Tropheryma whipplei*	–
36	*Moraxella catarrhalis*	–
37	*Streptococcus pneumoniae*	*Streptococcus pneumoniae*
38	*Haemophilus influenza* *Haemophilus parainfluenzae*	–
39	*Streptococcus pneumonia* *Haemophilus parainfluenzae*	–
40	*Moraxella catarrhalis* *Streptococcus pneumoniae*	*Moraxella catarrhalis*
41	*Haemophilus influenzae*	–
42	*Haemophilus influenzae*	–
43	*Haemophilus influenzae*	–
44	*Streptococcus pneumonia* *Haemophilus parainfluenzae*	–
45	*Streptococcus pneumonia* *Haemophilus parainfluenzae*	–
46	*Escherichia coli*	–
50	*Haemophilus parainfluenzae*	–
51	*Pseudomonas aeruginosa*	*Pseudomonas aeruginosa*
52	*Haemophilus influenzae*	–
53	*Haemophilus parainfluenzae*	–
54	*Haemophilus influenzae*	–
55	–	*Enterobacter cloacae*
56	*Haemophilus parainfluenzae*	–
59	*Haemophilus influenzae*	*Haemophilus influenzae*
60	*Haemophilus influenza* *Streptococcus pneumoniae*	–
63	*Haemophilus influenzae*	*Haemophilus influenzae*
64	*Haemophilus parainfluenzae*	–
66	*Haemophilus influenzae*	*Streptococcus pneumoniae*
67	*Haemophilus influenzae*	*Haemophilus influenzae*
68	*Haemophilus influenzae*	*Haemophilus influenzae*

-: means negative.

**Figure 3:**
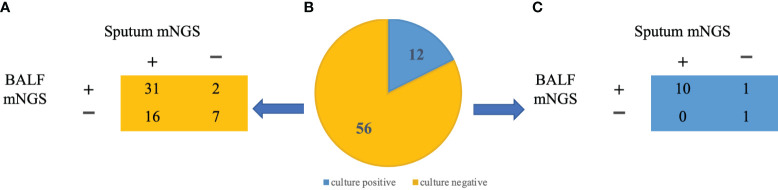
mNGS pathogen identification in BALF and sputum samples **(A)** Identification of pathogens in BALF and sputum samples in patients with negative culture results. **(B)** BALF culture results from 68 patients: 12 positives (blue), 56 negatives (yellow) **(C)** Identification of pathogens in BALF and sputum samples in patients with positive culture results.

In this study, we conducted a comparison between the outcomes of conventional microbiological culture and mNGS of BALF. Additionally, we evaluated the results of conventional microbial investigation of BALF in conjunction with paired sputum mNGS for the purpose of bacterial detection. (**Figure 3**) Among the 56 patients who had negative conventional BALF culture, only 7 individuals were negative for both BALF and sputum by mNGS. However, 31 patients were positive for both, suggesting that mNGS had a superior positive detection rate in comparison to conventional microbiological culture.

### Conventional microbial culturing for bacterial detection in sputum and BALF

The sputum of 68 patients was collected for conventional bacterial culture on admission; 14 (20.6%) and 12 (17.6%) patients had positive sputum and BALF culture results, respectively (*P* = 0.66; **Table 4**). The interval between the initial sputum collection and the BALF collection was three days (range: 3 to 13 days). The sensitivity, specificity, PPV, and NPV of the initial sputum culture to predict BALF were 0.42, 0.84, 0.36, and 0.87, respectively. The Kappa value was 0.24 (95% CI: −0.034 to 0.514, *P* < 0.047), indicating low agreement (**Table 5**).

**Table 4:** Bacterial culture results of sputum and BALF.

Patient number	Bacteria in sputum sample	Delay (days) between sputum1 and BALF sampling	Bacteria in BALF sample
3	*Haemophilus influenzae*	10(earlier)	–
25	*Haemophilus influenzae*	3(earlier)	–
33	*Haemophilus influenzae*	3(earlier)	–
41	*Haemophilus influenzae*	3(earlier)	–
53	*Haemophilus influenzae*	3(earlier)	–
58	*Haemophilus influenzae*	7(earlier)	–
59	*Haemophilus influenzae*	4(earlier)	*Haemophilus influenzae*
63	*Haemophilus influenzae*	3(earlier)	*Haemophilus influenzae*
14	*Streptococcus pneumoniae*	4(earlier)	*Streptococcus pneumoniae*
27	*Streptococcus pneumoniae*	3(earlier)	–
37	*Streptococcus pneumoniae*	3(earlier)	*Streptococcus pneumoniae*
6	*Haemophilus parainfluenzae*	4(earlier)	–
49	*Moraxella catarrhalis*	3(earlier)	–
55	*Enterobacter cloacae*	4(earlier)	*Enterobacter cloacae*
9	–	3(earlier)	*Moraxella catarrhalis*
67	–	6(earlier)	*Haemophilus influenzae*
68	–	3(earlier)	*Haemophilus influenzae*
66	–	3(earlier)	*Streptococcus pneumoniae*
31	–	3(earlier)	*Moraxella catarrhalis*
40	–	3(earlier)	*Moraxella catarrhalis*
51	–	13(earlier)	*Pseudomonas aeruginosa*

–: means negative.

**Table 5:** Number of pathogens in sputum and BALF by conventional microbial test in LRTIs patients.

Pathogen	BALF	Sputum	Sputum+/BALF+	Sputum–/BALF+	Sputum+/BALF–	Sputum-/BALF–
*Haemophilus influenzae*	4	9	2	7	2	57
*Moraxella catarrhalis*	3	1	0	3	1	64
*Streptococcus pneumoniae*	3	3	2	1	1	64
*Haemophilus parainfluenzae*	0	1	0	0	0	0
*Enterobacter cloacae*	1	1	1	0	0	67

+: means positive; –: means negative.

## Discussion

The identification of a causative pathogen of an LRTI in children is crucial to ensure administration of appropriate treatment, but suitable specimen selection and implementation of appropriate detection methods has always been a clinical challenge. The most prevalent clinical specimens for respiratory tract infections are sputum samples, which are easier to obtain than BALF samples. However, their diagnostic value is debatable. Previous studies have primarily assessed the differences in predilection between the upper and lower airways for infections with certain viruses (Soccal et al., 2010; López Roa et al., 2012; Bogoch et al., 2013; Wurzel et al., 2013; Hakki et al., 2014; Lachant et al., 2017; Boonyaratanakornkit et al., 2020) or bacteria (Dubourg et al., 2015; Lu et al., 2017). Most of the studies have been performed in unique patient populations, such as those who are immunocompromised due to immunodeficiencies (Azadeh et al., 2015), cystic fibrosis (Burns et al., 2012), or those who have undergone transplantation requiring immunosuppressants (Hakki et al., 2014; Boonyaratanakornkit et al., 2020). Thus, guidance for patients with ordinary respiratory tract infections is limited. mNGS has the potential to detect all infections in a clinical sample (Diao et al., 2022). Therefore, to the best of our knowledge, we present the first study to comprehensively assess the diagnostic usefulness of mNGS to detect bacteria, viruses, fungi, and mycoplasmas in sputum samples from children with LRTIs.

Tao et al. (Tao et al., 2022) reported that mNGS detected pathogens in BALF and sputum at rates of 82.7% and 75.8%, respectively, which were lower than the pathogen detection rates in our study (BALF: 94.1%; sputum: 98.5%). Furthermore, our detection rates were higher than those previously reported in adult patients (Duan et al., 2021; Diao et al., 2022). Moreover, the co-infection rates in BALF and sputum were high in our study, reaching 80.9% in sputum and 66.2% in BALF, which were again higher than that previously reported (53.3%) (Tao et al., 2022). The overall pathogen detection rate and mixed infection rate may have been higher in our study as we only included a small number of participants with a definite diagnosis of LRTI. Additionally, while some patients had unspecified pathogens, other had definitive ones, such as *M. pneumoniae* pneumonia, with solid lung lesions which required bronchoscopy. Thus, these selection biases likely influenced the pathogen detection rates in this study.

A meta-analysis revealed that conventional molecular methods identified one or more respiratory viruses in 55% of pediatric pneumonia cases worldwide. Children under the age of five years had the greatest rate of pediatric community-acquired pneumonia with a confirmed respiratory virus (Pratt et al., 2022). In our study, the viral detection rate using mNGS was 73.5% for sputum and 69.1% for BALF, which is higher than previous reports. Identification differences of viruses in upper and lower airway specimens have been discussed in several previous works (Renaud et al., 2013; Wurzel et al., 2013; Hakki et al., 2014; Azadeh et al., 2015; Lu et al., 2017; Boonyaratanakornkit et al., 2020). For example, a retrospective study found that the PPV and NPV of viral detection in upper airway specimens for the lower respiratory tract were 86% and 94%, respectively, suggesting that the upper airway detection results can accurately reflect the causative pathogen of a lower respiratory tract infection to a high degree (Hakki et al., 2014). Another study of patients who were immunosuppressed also found a high concordance between upper airway viral testing and BALF (PPV: 88%, NPV: 89%, Kappa = 0.729) (Lachant et al., 2017). Our study found that the overall PPV and NPV for viruses were 0.82 and 0.67, respectively, which is lower than previous reports. Further, the agreement was moderate (Kappa = 0.46). However, the concordance of sputum and BALF was significantly dependent on the identification of the virus. RSV ranked foremost among viruses detected in sputum and BALF, which is consistent with prior studies (Shi et al., 2015). In our study, RSV detection was highly concordant in the upper and lower airways (Kappa = 0.8), which is similar to the study by Boonyaratanakornkit et al. who investigated respiratory infections in hematopoietic stem cell transplant patients (92% concordance) and by Hakki et al. (100% concordance) (Hakki et al., 2014; Boonyaratanakornkit et al., 2020). These studies imply that in cases of positivity of upper respiratory tract testing for RSV, in combination with clinical and imaging evidence, that RSV infection of the lower respiratory tract should be considered.

In our study, human rhinovirus (HRV) detection was much higher in the sputum samples than in the BALF samples. Consistent with earlier findings (Wurzel et al., 2013), all positive BALF specimens were detectable in sputum specimens; however, there were five instances of a positive sputum sample with a negative BALF sample. Another study found a significant prevalence of HRV discordance in the upper and lower airways, with a Kappa value of 0.199 (Boonyaratanakornkit et al., 2020). Furthermore, previous studies have found that HRVs are more likely to replicate at colder temperatures, making the nasopharynx and upper airways more susceptible to infection (Mosser et al., 2005; Killington et al., 1977).

Two studies reported a high inconsistency rate between the upper and lower airways regarding ADVs, with Kappa values of 0.001 (Boonyaratanakornkit et al., 2020) and 0.213 (Luo et al., 2021). However, other studies have drawn opposite conclusions, reporting that upper airway specimens had a sensitivity of 69% and a specificity of 90% for detecting ADV and a moderate agreement (Kappa = 0.561). Furthermore, another study reported that ADV has a high specificity in children younger and older than three years of age (Lu et al., 2017). Herein, we found a low ADV positivity rate; however, all BALF-positive patients were also sputum-positive. Therefore, a negative upper airway test suggested that lower airway ADV isolation was unlikely, while a positive result did not consistently predict infection.

Few studies have documented the variations in the detection rates of bacteria in the upper and lower airways. Luo et al. (Luo et al., 2021) studied the consistency of bacteria detection rates in nasopharyngeal aspirate and BALF samples in 533 patients; however, the Kappa value was only 0.013, suggesting that the consistency between the two for bacterial detection was very poor. However, that study included patients who had already taken antibiotics on admission, which reduces the specimens’ positivity rate and indirectly affects the detection ability. However, two other studies reported good consistency between bacteria detected in the upper and lower airways, with Kappa values of 0.813 and 0.767 (Rong et al., 2020; Shi and Zhu, 2021). Therefore, they concluded that sputum samples should be given precedence in clinical practice under specific circumstances. Nonetheless, the participants in these two studies were adults. Thus, further study is required to see if this result can be applied to children. In our study, every patient underwent antibiotic treatment before undergoing bronchoscopy. Surprisingly, when we analyzed the conventional bacterial culture results of BALF, we found that only 17.6% of cases tested positive for bacterial presence. In contrast, the bacterial positivity rate of mNGS was significantly higher at 64.7%, showing a clear statistical difference. Notably, mNGS exhibited a higher capability to detect bacteria even in the presence of antibiotic therapy, aligning with findings from earlier research (Diao et al., 2022). While the total incidence of bacterial consistency in the upper and lower airways was low (Kappa = 0.3) in our study. Therefore, doctors should proceed with caution when diagnosing LRTIs based on bacteria identified in the sputum.

*M. pneumoniae* is a common pathogen that causes community-acquired pneumonia in children (Kutty et al., 2019), accounting for 10–40% of cases (Jain et al., 2015). Luo et al. (Luo et al., 2021) reported moderate consistency for *M. pneumoniae* detection between upper airway and BALF samples (Kappa = 0.407), implying that *M. pneumoniae* positivity from nasopharyngeal samples could be evidence of an *M. pneumoniae* infection. In our study, *M. pneumoniae* detection in the upper and lower airways were highly consistent, despite possible selection bias due to a limited sample size. This result also implies that in patients with suspected pneumonia caused by *M. pneumoniae* infection, that further testing for an upper airway infection with *M. pneumoniae* may be used to confirm the diagnosis.

The number of identified fungal cases was limited in our study; thus, a consistency analysis was not performed. However, *P. jirovecii* and *C. albicans* were highly consistent in the distribution of the four detected fungi in the upper and lower airways, despite the limited sample size. Hence, further research is needed.

There are some limitations to this research. First, selection bias is possible since this was a single-center study, and the expense of mNGS testing restricted the sample size. Furthermore, all patients received antibiotics prior to bronchoscopy, and after three days of empirical treatment, none of the patients showed signs of improvement. Therefore, whether our results apply to most patients with LRTIs requires confirmation in a prospective multicenter study using a larger sample of patients. Second, the mNGS-detected pathogens were not confirmed by other molecular tests on a genetic level due to the limitations of traditional techniques to detect infections. Lastly, the research period was only seven months, which does not represent differences in the incidence of various diseases, particularly viruses, across seasons, nor does it accurately reflect the total incidence of viruses.

## Conclusion

Viral detection in sputum and BALF samples were in moderate concordance; thus, clinicians can reliably diagnose lower respiratory tract viral infections based on sputum. However, such decisions should also consider underlying patient characteristics, clinical presentation, and the specific pathogen. Conversely, clinicians should cautiously diagnose a bacterial infection of the lower respiratory tract based on sputum samples only since bacterial identification was inconsistent and the concordance of results between BALF and sputum samples was poor.

## Data availability statement

The datasets presented in this study can be found in online repositories. The names of the repository/repositories and accession number(s) can be found below: The data presented in this study are deposited into CNGB Sequence Archive (CNSA) of China National GeneBank DataBase (CNGBdb) with accession number CNP0004464.

## Ethics statement

The requirement of ethical approval was waived by the ethics committee of The Third Affiliated Hospital of Zhengzhou University for the studies involving humans because This study was approved by the ethical committee. The studies were conducted in accordance with the local legislation and institutional requirements. Written informed consent for participation was not required from the participants or the participants’ legal guardians/next of kin because The written informed consent was provided.

## Author contributions

Conception and design: RS, YX, CZ. Acquisition, statistical analysis or interpretation of the data: SWZ, DZ, XD, WG, YW, SJZ, YZ. Drafting of the manuscript: YX, CZ. All authors reviewed and approved the final version of the manuscript. All authors contributed to the article and approved the submitted version.
